# A Realist Review of Violence Prevention Education in Healthcare

**DOI:** 10.3390/healthcare9030339

**Published:** 2021-03-17

**Authors:** Sharon Provost, Maura MacPhee, Michael A. Daniels, Michelle Naimi, Chris McLeod

**Affiliations:** 1 Interdisciplinary Studies, University of British Columbia, Vancouver, BC V6T 1Z4, Canada; 2 School of Nursing, University of British Columbia, Vancouver, BC V6T 1Z3, Canada; maura.macphee@ubc.ca; 3 Sauder School of Business, University of British Columbia, Vancouver, BC V6T 1Z2, Canada; michael.daniels@ubc.ca; 4 School of Population & Public Health, University of British Columbia, Vancouver, BC V6T 1Z3, Canada; michelle.naimi@gmail.com (M.N.); chris.mcleod@ubc.ca (C.M.)

**Keywords:** healthcare, patient violence, violence prevention education effectiveness, realist review

## Abstract

Violence from patients and visitors towards healthcare workers is an international concern affecting the safety and health of workers, quality of care, and healthcare system sustainability. Although the predominant intervention has been violence prevention (VP) education for healthcare workers, evaluating its effectiveness is challenging due to underreporting of violence and the inherent complexity of both violence and the health care environment. This review utilized a theory-driven, realist approach to synthesize and analyze a wide range of academic and grey literature to identify explanations of how and why VP education makes a difference in preventing violence and associated physical and psychological injury to workers. The review confirmed the importance of positioning VP education as part of a VP strategy, and consideration of the contexts that influence successful application of VP knowledge and skills. Synthesis and analysis of patterns of evidence across 64 documents resulted in 11 realist explanations of VP education effectiveness. Examples include education specific to clinical settings, unit-level modeling and mentoring support, and support of peers and supervisors during violent incidents. This review informs practical program and policy decisions to enhance VP education effectiveness in healthcare settings.

## 1. Introduction

Occupational violence refers to abuse, threats, intimidation, or assault of individuals in the course of their work [1], and is commonly categorized according to the relationship between the perpetrator and worker [2]. There may be no relationship such as in the case of violence related to crime (type I), a client/provider relationship (type II), a peer or work relationship (type III), or a personal relationship such as domestic violence brought into the workplace (type IV) [2].

Although type II workplace violence exists in many industries, including law enforcement, education, and retail services, healthcare workers suffer proportionately more type II violence than workers in any other sector [3,4,5] and have the highest percentage of violence-related injury claims [6]. A recent meta-analysis from 30 studies found that one in four healthcare workers experience physical violence by patients or visitors every year [7]. As evidenced in studies and literature reviews, type II violence from clients, patients and long term-care residents towards healthcare staff such as nurses, doctors, and care aides is an increasing issue that affects the wellbeing of healthcare workers and their ability to provide care [8,9,10,11,12,13,14]. Violence towards healthcare workers is a documented concern not only in the authors’ jurisdiction [15,16,17], but across many countries, as acknowledged by international organizations [9,11,18].

Type II violence in healthcare can be physical, including kicking, hitting, biting, pushing, sexual assault, stabbing, and assault with a weapon, or psychological, such as threats of immediate or future physical force or attack and verbal abuse or harassment [18]. Multiple contexts influence the risk of violence towards healthcare workers, including physical environments, policies, characteristics of populations served, individual patient disease processes, the nature of the patient/caregiver interactions, and consumer frustration with system issues, such as wait times or restrictive policies [8,19,20,21,22]. In particular, healthcare workers in emergency departments, psychiatry, and residential (long-term care) areas have been documented as being at high risk for type II violence [23,24].

Measuring the prevalence of type II violence in healthcare is challenging, as violence is chronically underreported due in part to differing interpretations of what behaviours constitute violence [17,20] and to the normalization of violence as ‘just part of the job’ and not worth reporting [10,20]. A published review of prevalence studies reported that 22% to 90% of healthcare workers have experienced verbal abuse, 12% to 64% experienced threats of physical violence, and 2% to 32% have suffered an actual assault [21].

The predominant intervention to address type II violence in healthcare is violence prevention (VP), which is administered through programs that teach healthcare workers how to prevent, manage, and report violence [10,22,25,26,27]. The curriculum of VP programs typically includes instruction on communication skills and de-escalation of aggression, and some programs also focus on defining and assessing the risk of violence, emotional self-management, and self-defense [20,28,29,30]. Delivery of VP education can involve online modules, classroom training sessions, or a hybrid of both, and can vary in length from one hour to multiple days [22,27,30,31]. As such, although VP education is extensively used as an intervention to address type II violence in healthcare, its content, context, and method of delivery vary widely.

Despite the frequency of its use, determining the overall effectiveness of violence prevention education has been challenging [21,22]. Changes in violence incidence rates may represent effects of the education, external factors, new patterns of reporting, or a combination of all three [21]. Traditional reviews of VP program evaluations are generally inconclusive regarding the efficacy of these programs, citing the variety of content across programs, underreporting of violence, and complexity of healthcare settings as major barriers [22,27,32,33,34]. To address some of these challenges, this review utilizes a realist approach, a method gaining in popularity that helps to synthesize evidence from the literature to provide findings and recommendations that are practical and useful for healthcare providers and administrators—the intended audience.

Developed in the United Kingdom by Pawson and Tilley [35], a realist review offers the ability to provide insights into the effectiveness of a program in ways that systematic or narrative reviews typically cannot: by utilizing information from existing literature to explore how and why interventions involving human behaviour succeed or fail [36]. Educational programs such as violence prevention which aim to change participant attitudes and actions are designed with an underlying theory of how participants will learn and apply the content [37]. Unlike a typical literature review that summarizes or synthesizes findings from different studies, a realist approach begins with a theory of how an intervention is intended to work and then seeks information from a wide range of literature to support, refine, or refute that initial theory [38]. The resulting program theory is described using realist explanations, known as context-mechanism-outcome (CMO) configurations “that spell out the relationship between particular features of context, particular mechanisms and particular outcomes” [39] (p. 13). As illustrated in the findings of this review, it is the context—such as the type of educational content or the format of delivery—which is necessary to trigger an underlying mechanism (e.g., participant’s confidence) that leads to intended outcomes (e.g., the application of VP skills). The realist approach is useful to policymakers who want to know what contextual factors are most likely to produce their desired outcomes, particularly with complex social and healthcare programs, where many interventions are possible [38] (further definitions are available in the Supplementary Materials: Table S1: Glossary of terms).

Findings from the review are intended to provide practical information and recommendations through “generalizable knowledge about how context shapes the causal mechanisms through which a programme produces its outcomes” [40] (p. 113). This review of violence prevention education was designed to answer three questions:

- For whom is VP education likely to be effective?
- What are the underlying mechanisms by which VP education results in the intended outcomes?
- In what contexts/circumstances does VP education contribute to effective violence prevention and management practices?

## 2. Methods

This review was conducted between February 2018 and January 2019 with two researchers working together (February to May 2018, S.P. and M.M.P.; August 2018 to January 2019, S.P. and M.N.). The review followed the RAMESES publication standards for realist reviews [41], and the methods used are presented in the five steps delineated by Pawson [37]: (1) identifying the initial program theory, (2) searching for evidence, (3) selecting documents, (4) extracting and organizing data, and (5) synthesizing evidence and drawing conclusions. The RAMESES publication standards for realist synthesis are internationally recognized standards for realist methods, similar to CONSORT standards for conducting randomized controlled trials [42].

### 2.1. Step 1: Identifying the Initial Program Theory

An underlying premise of a realist research approach is that no intervention is entirely new, and developing a theory of how and why a program or policy works builds upon existing knowledge about similar programs [43]. A scoping review of the literature contributed to the development of an initial VP education program theory for this study. The scoping of the literature was conducted using the search function of the University of British Columbia (UBC) Library and through Google Scholar using the terms “violence prevention education,” “healthcare violence prevention,” and “type II violence in healthcare.” These searches revealed a large body of literature predominantly focused on prevalence in different jurisdictions and settings, contributing factors for violence, and effects of violence [44,45,46,47]. A lesser but still significant number of academic and grey literature publications described healthcare workers’ experiences and/or the impact of violence [48,49]. Some articles reported on individual interventions and programs [50] or evaluations of an intervention [25,51,52]. A small number of systematic reviews of interventions and their effectiveness were identified [22,27,28,53,54]. Scoping of the literature confirmed a knowledge gap regarding the effectiveness of VP programs and identified 11 articles that offered ideas for an initial program theory (Supplementary Materials: Table S2: Initial program theory)

### 2.2. Step 2: Searching for Evidence

Where systematic reviews establish and do not change inclusion/exclusion criteria for literature searches, a realist review approach is iterative with criteria for further searches evolving as a program theory is developed and tested [36,38,55]. The search strategy for this review was refined with the assistance of an information specialist. Four academic databases within EBSCOHOST were identified to encompass healthcare and other social sector disciplines where type II violence is prevalent: CINAHL (nursing; healthcare), MEDLINE (medicine), ERIC (education), and PsychInfo (psychology and social work). The three key search concepts were workplace violence, healthcare, and prevention education, and the search was limited to the English language with full-text access. The electronic functionality within each database was used to guide the search terms used. Preliminary searches were used to identify timeframes for each database when the most potentially relevant literature was published: 10 years (2008–2018) for MEDLINE, ERIC, and PsychInfo, and 20 years (2000–2018) for CINAHL. As several salient articles found in the scoping review were not returned in the EBSCOHOST CINAHL search, additional snowball searches were conducted in CINAHL, Google Scholar, and Scopus. Google Scholar and Mendeley publication alerts were set to identify relevant newly published literature.

### 2.3. Step 3: Selecting Documents

Criteria for document screening in realist reviews are based on (1) how well the document informs the research questions (relevance) and (2) the trustworthiness of the research method used to arrive at the document’s conclusions (rigour) [55,56]. In all stages of screening, work was shared between two researchers (S.P. and M.M.P.), with the first 10 documents screened together to ensure a consistent approach. Formal criteria were used to screen documents at three levels: title, abstract, and full document (Supplementary Materials: Figure S1: Searching and screening strategy).

Of the 1656 documents from the initial searches, 1173 documents were identified as not relevant based on title screening, and 472 documents were retained that focused on type II violence and violence prevention in healthcare or other social sectors. During the abstract screening phase, a further 289 documents were excluded based on relevance, and 194 documents were retained. Five additional articles from snowball searches were added to the 194 abstract screened documents for full article screening for relevance and rigour. Of the 199 documents full article screened, 149 were excluded as not relevant to informing the research questions, and 50 were identified for inclusion. Iterative searches conducted during analysis contributed an additional 14 documents for a total of 64 included documents in the review. A list of all 64 documents included in the review is available in the Supplementary Materials (Table S4: Included documents).

### 2.4. Step 4: Extracting and Organizing Data

In qualitative research, words or terms are often used as “codes” to capture the essence or meaning of information [56], which are then linked and structured into meaningful data [57]. In a realist review, data are coded as whole or partial CMO explanation threads instead of separately as contexts, mechanisms, and outcomes [58]. A deductive coding framework was created in NVivo© using the initial program theory. In addition to this deductive coding approach, new codes were identified and included in the coding framework as they emerged (i.e., inductive coding). Coding was shared between two researchers (S.P. and M.N.) and an iterative cycle of discussion, writing of memos, data reorganization, and further literature searches was used to continually refine the initial program theory.

### 2.5. Step 5: Synthesizing Evidence and Drawing Conclusions

Two researchers used a cyclical process of reviewing text segments for potential CMO explanations and identifying links between the CMOs and the program theory. CMO mechanisms are often associated with mid-range or substantive formal theories from fields such as psychology and sociology. These theories provide support for causal mechanisms linking contexts and outcomes within a program theory. During this final step, the researchers identified the following theories associated with this program theory’s mechanisms: self-efficacy theory [59] and risk of learning theory [60] (Supplementary Materials: Table S3: Supporting formal theory)

The practical purpose of a realist review requires that researchers “transform weighty reports and detailed analysis into accessible formats that speak to the context in which the findings will be used” [61] (p. 183). Through an iterative process of coding and refinement, the final product was a refined program theory on violence prevention education comprised of 11 key CMO explanations.

## 3. Results

### 3.1. Search Results

Screening and selection of documents from the initial and iterative searches resulted in 64 documents from the peer-reviewed and grey literature, including articles, reports, policy protocols, editorials, and book chapters (Supplementary Materials: Table S4: Included documents)

### 3.2. Focus of the Review

The findings in this review address the research questions of how, why, and in what contexts violence prevention education is effective; however, insufficient evidence was found to answer the question of for whom. An overarching finding from this review of violence prevention literature is a confirmation that VP education is embedded in a larger VP strategy and that evaluation requires consideration of both the VP education program and the factors that influence successful knowledge and skill application in practice settings [20]. Consequently, the 11 CMO explanations of program effectiveness identified as most salient involve contexts before, during, and after formal education. The complete refined program theory with the CMO explanations organized across this time continuum is accessible in the Supplementary Materials (Figure S2: Review program theory for violence prevention education). The following sections describe and illustrate the 11 CMO explanations in relation to three important outcomes: decreasing violent incidents, decreasing violence-related injuries, and increasing violence incident reporting. Each explanation is supported with quotes from documents included in the review.

### 3.3. CMO Explanations 1–6: Decreasing Violent Incidents

**Figure 1:**
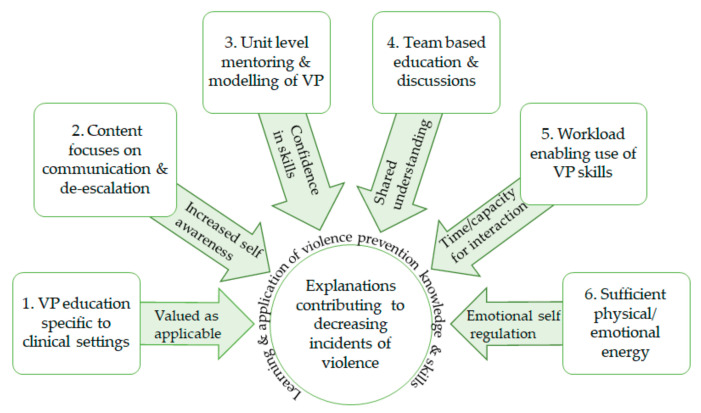
Context-mechanism-outcomes (CMOs) 1–6: explanations decreasing violent incidents. VP: violence prevention.

Six CMO explanations contribute to decreasing violent incidents: VP education specific to clinical settings, content focused on communication, unit-level mentoring, team-based education and discussions, workload enabling meeting patient needs, and sufficient physical/emotional energy. (Figure 1).

#### 3.3.1. CMO 1: VP Education Specific to Clinical Settings

When content is specific to participants’ clinical settings (C), they are more likely to value (M) the education as applicable and engage in learning (O).

Valuing education as applicable involves two contextual factors: the content taught and—to a lesser degree—by whom. Participants perceive education as worth their attention when the content is specific to their clinical practice setting and content cases or examples reflect the workplace violence they experience. Additionally, when facilitators or trainers provide examples from their own clinical experiences with workplace violence, participants value the content and see it as more applicable.

*“Participants in the workplace violence program were taught information that was directly applicable to their work environment. The tabletop exercise provided contextual meaning by using video case studies that were both realistic and applicable to the environment in which the acquired knowledge would be applied”*

[62] (p. 471)

*“…ward specific training may address these limitations by facilitating the transfer of knowledge to practice, developing skills identifying problems and implementing prevention strategies”*

[63] (p. 7)

*“Someone who teaches aggression management should be on the wards to get the feel of what actually happens’ (Ward security staff member). ‘Have the trainer experience the ward environment and apply the program to the situations on the ward’ (Nursing staff member)”*

[64] (p. 237)

#### 3.3.2. CMO 2: Content Focuses on Communication and De-Escalation

When education focuses on communication and de-escalation skills (C), participants have increased self-awareness (M) and are more likely to use a VP approach to prevent escalation and violence (O).

VP education that focuses training on effective communication and de-escalation skills increases participants’ awareness of how their own emotions and approach in interactions with patients can influence the risk of violence. When participants are more self-aware, they are more likely to use communication and de-escalation skills, which may prevent violence. Participants’ focus on prevention may be diminished when training sessions introduce self-protection ‘break away’ maneuvers: ways in which healthcare workers can free themselves more easily if their arm, hair, or clothes are grabbed by patients. Although break-away techniques may provide a temporary sense of confidence in managing violence, studies indicate that limited or brief training in these methods is less effective, with few individuals utilizing these types of skills in practice [65,66].

*“Direct skills teaching [provides] knowledge of behavioural skills and strategies for emotional regulation [leading to] increased confidence/self-efficacy [and] enhanced interpersonal style when managing aggressive behaviour [and] emotional regulation when faced with aggressive behaviour”*

[33] (p. 237)

*“Crucially, training needs to help staff understand how problems, such as CB [challenging behaviour], can arise within and as a result of their routine interactions with clients”*

[67] (p. 237)

*“…training helped them control their temperament in a challenging environment and also enabled them to effectively practice active listening and empathy”*

[68] (p. 297)

*“…training interventions that enhance staff communication skills do decrease violent incident rates”*

[27] (p. 2828)

#### 3.3.3. CMO 3: Unit Level Mentoring and Modeling of VP

Unit level mentoring and modeling of VP (C) increases confidence (M), resulting in increased use of VP approaches (O).

Participants’ attainment of knowledge and skills from VP education is influenced by their educational experience and by how their learning is reinforced and supported in their workplace. When participants see role modeling of VP skills and have access to expert advice and mentoring, they have more confidence to apply VP knowledge and skills in their practice.

*“It is recommended that early contact is made with clinical experts when high-risk patients are first identified, rather than following an incident, and that key ward staff are trained and mentored to develop confidence in managing patients with a risk for violence/aggression”*

[63] (p. 13)

*“Experienced workers can mentor and guide less experienced colleagues in communication and care delivery strategies that may calm patients and visitors, diffuse tense situations”*

[69] (pp. 182–183)

*“Because of the sometimes impromptu nature of violence, consequent debriefing and the sensitivities involved, a change agent from within the clinical team may have been more successful as an internal ‘implementer’ working with peers”*

[70] (p. 12)

#### 3.3.4. CMO 4: Team-Based Education and Discussions

Team-based VP education, reinforced with regular team discussions of violence incidents (C), increases teams’ shared understanding (M), supporting a consistent approach to prevent and manage violence (O).

Violence prevention activities that foster team interaction increase the team’s ability to respond effectively as a unit to situations of potential violence. Training as a team and learning from team-based discussions about violence and debriefing of incidents increases participants’ shared knowledge, optimizing consistent approaches to workplace violence [60].

*“Wards adopting a whole-team approach are more likely to reduce the risk of assault than individual advances in knowledge and skills… Clinical managers should not only ensure that sufficient numbers of their staff are trained, but also that as many staff as possible are trained together at the same time, to foster such approaches and facilitate maximal gain”*

[33] (p. 453)

*“Interventions to support nurses and nursing teams in processing transgressive behaviour in care relationships should be implemented on a team level, incorporating the culture of the ward and the dynamics of teams”*

[71] (p. 2381)

*“Participants in the intervention group of a structured program for regular discussion of workplace violent incidents reported an improved awareness and management skills”*

[10] (p. 21)

#### 3.3.5. CMO 5: Workload Enabling the Use of VP Skills

When workload enables sufficient time with patients (C), participants have the opportunity to apply VP knowledge and skills (M), decreasing the risk of violence.

A limiting factor in the application of violence prevention knowledge and skills is the availability of healthcare workers; time and workload capacity. When workload is reasonable, workers have the required time with patients to interact and communicate, observe behaviour, and apply VP knowledge and skills that prevent or de-escalate aggressive behaviours.

*“Most ED RNs thought that the classes they were forced to take were not effective or had little efficacy in successfully de-escalating patient behaviours. Most ED RNs cited a lack of time to implement the tools taught in these classes”*

[72] (p. 549)

*“Staff members also identified barriers that sometimes prevented their managing behaviour problems optimally. These included time pressure”*

[73] (p. 38)

*“Training alone is not enough and staff need to be enabled to learn with adequate support and resources e.g., … reasonable workload to apply skills and communicate with patients or residents”*

[74] (p. 20)

#### 3.3.6. CMO 6: Sufficient Physical/Emotional Energy

When healthcare workers have sufficient physical and emotional energy (C), they have an increased ability to self-regulate their emotions (M) and use VP approaches (O).

An individual healthcare worker’s physical and emotional state affects their ability to self-regulate their emotions and behaviours. Individuals who are stressed by personal life factors, poor working relationships with peers or supervisors, or from dealing with frequent violence, are less able to self-regulate and prevent violence.

*“Participants are more able to apply skills when they are fresh and have energy early in their shift but when they are tired and nerves are frayed they resort to previous behaviour”*

[72] (p. 550)

*“The high levels of physical, verbal and sexual violence combined with the structural violence of caring in an understaffed and under-resourced environment stretches workers to the limit. Personal support workers leave physically and mentally exhausted”*

[74] (p. 21)

### 3.4. CMO Explanations 7–10: Decreasing Injuries from Violence

**Figure 2:**
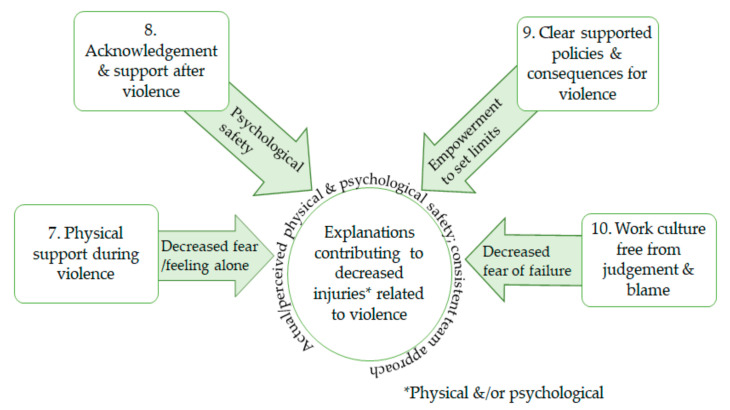
CMOs 7–10: explanations decreasing worker injury from violence.

Four CMO explanations were identified that contribute to decreasing physical and psychological injuries for healthcare workers: support during violence, support after a violent incident, clear and supported violence policies, and a nonjudgmental and blame-free work culture. (Figure 2)

#### 3.4.1. CMO 7: Physical Support during Violence

Physical support from supervisors and peers during violence (C) decreases fear and feelings of being alone (M), increasing actual or perceived physical safety (O).

Experiencing verbal and physical violence from patients can be traumatic and create fear for personal safety at work. When healthcare workers can rely on peers and supervisors to be physically present and available to provide support during an incident of violence, they have less fear for their physical safety and feel less alone.

*“’It feels great to have support at times like these. It helps me feel like I am not alone when these situations occur and that someone has my back’”*

[75] (p. 125)

*“A sense of abandonment underlay accounts where a physical absence of support staff and managers on the wards meant that staff ‘often felt totally alone in a difficult and dangerous situation’”*

[76] (p. 5)

*“The RN who steps in and either takes over for an RN who is experiencing a challenging patient or intervenes for another nurse who might be newer or more timid… ‘ We have a couple of nurses who just stand up, you know, for the weaker nurses who can get picked on by certain patients. They will just slip in and take over the assignment or whatever they can do to help, but in a positive way’”*

[72] (p. 552)

#### 3.4.2. CMO 8: Acknowledgement and Support after Violence

When individuals receive acknowledgement and non-blaming emotional support from supervisors and peers after violence (C), they feel psychologically safer (M) and have less psychological injury from the violence (O).

When health care workers receive acknowledgement that violence occurred and feel supported instead of blamed by peers and leaders, they perceive the workplace as psychologically safe and are less likely to feel emotionally traumatized. Consistent with trauma experienced by victims of non-occupational violence [77], when a healthcare worker does not receive the support they expect, they may experience a secondary psychological trauma, potentially more damaging than the trauma from the violent event.

*“More importantly nurses in the study felt most supported when the manager acknowledged the event as explained by this RN, ‘just having the event recognized as something that was critical and you know, it was traumatic and …they weren’t minimizing it and actually embracing it as something that was not acceptable’”*

[78] (p. 7)

*“The nature of the organisational response to the traumatised staff member can therefore play a pivotal role in the process of recovery and, where the organisational response fails to understand or consider the needs of the victim(s) can itself constitute a source of secondary injury or trauma”*

[79] (p. 481)

*“Participants actively looked toward their colleagues and managers for support and acknowledgment following client violence in the workplace, and indeed having supportive peers and supervisors can significantly improve a victim’s sense of coping and lessen their fear of further attacks”*

[80] (p. 293)

*“Many other nurses described feeling very angry, unsupported and blamed by their managers. Some RNs never heard from their managers following events of patient violence, while others described receiving a phone call or a brief conversation, which was felt to be thoughtful, but not sufficiently supportive”*

[78] (p. 7)

#### 3.4.3. CMO 9: Clear, Supported Policies and Consequences for Violence

Clear, supported policies with consequences for violence (C) empowers healthcare workers to set limits (M), resulting in a greater ability to manage aggression and violence (O).

When policies relating to violence are clear, visibly supported by leaders and include reasonable consequences for violent behaviours from patients and visitors, healthcare workers feel empowered to set limits and enact policies to manage aggression, resulting in a more consistent approach and decreased risk for injury.

*“Organizational factors like clear expectations for patient behaviour and consequences empower management and staff members to feel less frustrated and more equipped to deal with violence”*

[72] (p. 549)

*“Zero tolerance policy enforcement is thought to be constructive in terms of supporting and empowering staff to have confidence in managing problematic patients and hostile situations”*

[81] (p. 97)

*“An organization that positively addresses violence through the themes of consistency, consequences, and collaboration potentially mitigates the development of cynicism and conflict as maladaptive reactions of staff”*

[49] (p. 15)

#### 3.4.4. CMO 10: Work Culture Free from Judgement

When the work culture is free from shame and blame (C), healthcare workers have less fear of failure and of being perceived as incompetent (M) and are more likely to apply new knowledge and skills to effectively prevent or manage violence (O).

A supportive unit culture where healthcare workers are not blamed or judged for errors decreases their fear of being perceived as incompetent and increases confidence to try new skills and report incidents that have occurred.

*“Staff may feel less confident in taking the risk to apply new skills if they fear that their image may be harmed and they are seen as ignorant or incompetent”*

[60] (p. 2)

*“Nurses are reluctant to report violence in the workplace and may not seek support after incidents of violence because they think asking for help may be interpreted as personal weakness or professional failure”*

[82] (p. 45)

*“When admitting (or simply calling attention to) mistakes, asking for help, or accepting the high probability of failure that comes with experimenting, people risk being seen as incompetent, whether in a narrow, particular domain, or more broadly. Reluctance to take such interpersonal risks can create physical risks in high-risk industries”*

[60] (p. 256)

### 3.5. CMO Explanation 11: Increasing Reporting

**Figure 3:**
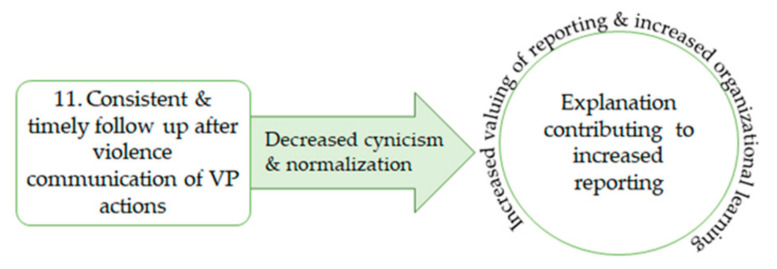
CMO 11: explanation increasing reporting.

The final CMO explanation relates to how the actions that are taken by leaders after violence influences whether staff will formally report future violence (Figure 3).

#### CMO 11: Follow up after Violence

When leaders provide consistent, timely follow-up and action to prevent further violent incidents (C), healthcare workers are less likely to perceive violence as normal (M) and are more likely to formally report violent incidents (O).

*“When staff do not see any result or change as a consequence of reporting violence when experience violence they feel hopeless and resigned that reporting is of no benefit and will not report”*

[47] (p. 271)

*“Registration of violent incidents without regularly scheduled, structured feedback discussions may have increased frustration in the control group, leading to less likelihood of reporting”*

[83] (p. 674)

*“Recordkeeping was rated as the second lowest subcomponent in terms of importance in reducing WPV. This finding may be related to employees not recognizing any benefit of recordkeeping as a form of WPV prevention”*

[84] (p. 381)

## 4. Discussion and Recommendations

The findings in this realist review answer the research questions regarding the mechanisms through which VP education works to attain the intended results and which contexts contribute to the program’s effectiveness. The review identified 11 CMOs that elucidate how (e.g., through shared understanding) and under what circumstances (e.g., when there are team discussions and teams are trained together) VP education is effective. However, the evidence was less clear regarding the question of for whom the education is most effective, which may relate to the complex nature of violence and heterogeneity of VP programs.

A realist approach to literature review addresses real-world issues through findings that “speak directly” to decisions regarding the creation or revision of social or health programs or policies [36] (p. 169). The 11 CMO explanations identified in this review translate into practical recommendations that guide actions to optimize violence prevention education. (Table 1). This review’s focus on explanations of how and why education is learned and utilized allows the findings to be applied to violence prevention education across different healthcare settings where contexts exist similar to those identified in the explanations [85].

Addressing workplace violence is a high priority within occupational health and safety, particularly during challenging times such as the current global COVID-19 pandemic [88]. Leaders in occupational health and clinical operations often have responsibility for large numbers of employees and experience increasing demands on their limited resources of time, effort and funding [89,90]. The findings and recommendations from this review provide specific information to guide the investment of resources to achieve the best outcomes [36] (Table 1). For example, the first CMO states that for individuals to engage with the education, the content needs to be specific to their clinical setting and the examples need to reflect the violence they experience. A traditional review might recommend that education needs to be relevant to participants. By contrast, the focus on understanding the how and why in a realist review provides a clearer picture of what ‘relevant’ looks like and what actions to undertake to achieve it.

The CMO explanations from this review are supported by evidence in the literature. Increased systematic reporting, as recommended in this review, and more rigorous mixed methods approaches to VP education evaluation will strengthen our knowledge of interventions to prevent and mitigate health workplace violence. The 11 CMO explanations and the program theory in this review serve as a foundation for future testing and refinement. As this review utilized secondary data from non-realist studies with different methodologies that asked different kinds of questions, the review may not have identified all of the relevant explanations of VP program effectiveness. As the search criteria included only English language documents, the findings may be less applicable to VP education in non-English speaking countries or cultures. Future research could address these limitations through the analysis of primary data from a realist evaluation of violence prevention education, and the application of a realist approach to literature in other languages.

## Figures and Tables

**Table 1:** Recommendations for violence prevention education.

Findings: CMO Explanations	Recommendations
1. VP education specific to clinical settings	□ Conduct education in clinical areas [63] □ Area-specific content and relevant examples [62,72,76] □ Trainers have knowledge of clinical area [64]
2. Focus on communication and de-escalation	□ Focus VP education on self-awareness, communication and de-escalation skills [27,68,76] □ VP sessions do not also include breakaway techniques [66]
3. Unit level VP modeling and mentoring	□ Create formal unit mentors/champions [69,70,86] □ Available VP advice from instructors e.g., consults, refreshers, and debriefing [63]
4. Team-based approaches to VP education	□ Train team members together for education and refresher activities [33,71] □ Promote team discussions about violence and VP [10,71]
5. Workload enabling use of VP education	□ Review and adjust workloads to allow time for violence risk assessment and use of de-escalation skills [20,74,86]
6. Sufficient physical and emotional energy	□ Supports for psychological workplace health (employee assistance support and counseling) [20,50] □ Ensure sufficient staffing and shift breaks [20]
7. Physical support during violence	□ Education includes supporting others during violence [76] □ Review physical layout, equipment, staffing levels, access to help, e.g., isolation of areas, alarms, and security [49]
8. Acknowledgement and non-blaming support after violence	□ Promote non-blaming support after violence [80] □ Education and guidelines for leaders/supervisors on how to support workers after violence [75,80,87]
9. Clear, supported VP policies	□ Revise policies/programs with worker involvement [49,50] □ Consistent implementation and support of VP policies through discussion, debriefing and monitoring [78] □ Educate leaders/supervisors on how to enact and supportviolence policies [31]
10. Work culture free from judgement or blame	□ Role model a non-blaming learning approach to follow up of all accidents and errors [87] □ Education and coaching to support a culture of safety approaches (i.e., non-blaming) [31]
11. Follow-up actions after violence	□ Provide guidelines and training for managers on violence follow-up including timeliness, communication, and preventative actions [31,75] □ Systematically monitor organizational violent events and follow up [20,21]

## Supplementary Materials

The following are available online at https://www.mdpi.com/2227-9032/9/3/339/s1. Table S1: Glossary of terms; Table S2: Initial program theory; Figure S1: Search and screening strategy; Table S3: Supporting formal theory; Table S4: Included documents; Figure S2: Review program theory for violence prevention education.
