# Supplementary material for: A Realist Review of Violence Prevention Education in Healthcare
Source: Healthcare (Basel). 2021 Mar 17;9(3):339. doi: 10.3390/healthcare9030339 (PMC8002651; doi:10.3390/healthcare9030339)
Supplement: Supplementary file 1 [file healthcare-09-00339-s001.pdf]

Table S1. Glossary of terms.

CMOs, CMO explanations or CMO configurations	a realist description of the contexts, mechanisms and outcomes used in Pawson and Tilley's [1] applied realism approach to review and evaluation that explain how and why programs and policies work
Context	the characteristics of both settings and individuals that can shape how individuals reason in response to programs or resources. Characteristics include - but are not limited to - existing policies, practices, social rules, norms, beliefs, attitudes, material and social structures, and interrelationships Social settings are open systems, and as such, contexts are not static and evolve over time. In addition to affecting the attitudes and reasoning of individuals, contexts can also affect whether the required resources are available to implement action towards the intervention's objective [2].
Initial program theory	the beginning theory about how a program or intervention is likely to work that forms the starting point for realist research
Mechanism	An underlying reasoning, belief system, process, or structure that operates in particular contexts to contribute to outcomes. Mechanisms are usually hidden, are sensitive to variations in context, and generate outcomes when they are activated [3]
Outcome	The result or effect of a program or policy such as violence prevention education. Outcomes can be intended or unintended, observable such as a behaviour, or less tangible such as increased confidence or knowledge. Outcomes close in time and space to the intervention may be referred to as "proximal" outcomes while longer-term objectives of the program can be described as distal and/or program outcomes [4].
Realism	philosophy regarding the nature of reality that posits that a real world exists but our experience of it is filtered through our senses, culture and language [5].
Realist evaluation	An evaluation of a policy or program using an applied realist approach to make transparent how, why, when and for whom an intervention is effective. A realist evaluation uses a similar process as a realist review but uses primary data as evidence.
Realist review or synthesis	A literature review using a realist approach. A realist review follows a similar process as a realist evaluation but uses secondary data as evidence.
Refined program theory	The theory expressed as CMO explanations about how, why and for whom a program or intervention works based on the evidence and analysis from a realist review or evaluation.
Substantive, formal or existing theory	"…Existing theories within particular disciplines…used to help understand interventions. For example, in the social sciences theories may deal with topics such as 'cognitive development', 'deviance control', or any of the wider ambitions of interventions." [6]

References for Table S1: Glossary of terms

[1] R. Pawson and N. Tilley, *Realistic Evaluation*. London: Sage, 1997.

[2] T. Greenhalgh *et al.*, "Resource and training materials for realist evaluation." p. 44, 2017.

[3] G. Westhorp, "Understanding Mechanisms in Realist Evaluation and Research," in *Doing Realist Research*, N. Emmel, Trisha Greenhalgh, A. Manzano, M. Monaghan, and S. Dalkin, Eds. London: SAGE, 2018, pp. 41–57.

[4] Indiana University, "Glossary of Terms," *Shaping Outcomes*, 2006. [Online]. Available: http://www.shapingoutcomes.org/course/glossary/index.htm. [Accessed: 06-Aug-2020].

[5] T. Greenhalgh *et al.*, "Philosophies and Evaluation Design • The RAMESES II Project," 2013. [Online]. Available: http://www.ramesesproject.org/media/RAMESES_II_Philosophies_and_evaluation_design.pdf. [Accessed: 23-Sep-2017].

[6] G. Wong, G. Westhorp, R. Pawson, and T. Greenhalgh, "Realist Synthesis. RAMESES Training Materials," *The RAMESES Project*, no. July 2013. RAMESES PROJECT, London, p. 55, 2013.

Table S2. Initial program theory*.

When /if	Then participants/ health care (HC) workers will	Resulting in
On- line modules mirroring the classroom content are completed prior to the classroom sessions(s)	learn and assimilate information through repetition and reinforcement	Increased knowledge of how to think about, prevent and manage violence
Violence scenarios are generated by participants from their own experience and used for discussion	learn information through envisioning how the content can be applied	Demonstration of the desired violence prevention, management and reporting actions
Information is given on the components and importance of risk assessment for violence	assume a belief that violence is not inevitable and can be prepared for and prevent violence	Violence risk assessment becoming part of normal care of patients
Leaders follow up with HC workers after violence to inform actions to prevent further violence	see reporting as worthwhile and have increased trust that actions will be taken	Communication of risk of violence and formal and informal reporting
Peers support each other during and after violence	trust that "someone has their back" and they are not alone	Increased feelings of safety, less fear and more confidence to try new VP skills
HC workers have a heavy workload, multiple demands and lack of time	likely feel overwhelmed and fatigued	Inability to apply knowledge to assess for, prevent and de-escalate violence
Thoughtful, non-blaming debrief sessions are held with HC workers after violent incidents on a routine basis	have reinforcement of the curriculum content applied to practice	Increased likelihood of integrating violence prevention into practice routine
Education focuses on self -awareness and participant role in communication to prevent violence	self reflect on previous interactions and potential future situations	increased demonstration of reflexive behaviour and self-management in violent situations
Participants are given definitions and examples of what constitutes violence	conceptualize and reframe violence to included unintended violence	Identification of violence from a new perspective and increased completion of risk assessment and reporting

*Developed from scoping of the literature and consultations with content experts prior to review.

Table S3. Supporting formal theory .

**Context**	**Mechanism**	**Formal Theory**
1. Education specific to clinical settings	↑ content valued as applicable to practice	
2. Education focussed on communication & de-escalation	↑ self-awareness of emotions & communication	
3. Unit level mentoring & modelling	↑ Confidence in VP skills	Self – efficacy theory (Bandura, 1982)
4. Team-based VP education & regular team discussions & decision making	↑ team trust & shared understanding	Team learning (A. Edmondson, 1999)
5. Workload enabling meeting patient needs	time/capacity for patient interaction	Job demand/ Resource Model (Bakker & Demerouti, 2007)
6. Sufficient physical/emotional energy	↑ self-regulation of emotions	
7. Physical support from supervisors & peers during violence	↓ fear for personal safety & ↑ confidence to manage violence	Secondary psychological injury (Canadian Resource Centre for Victims of Crime, 2005)
8. Acknowledgement & non blaming support from manager peers post violence	↑ perception of psychological safety	Risk of learning (A. C. Edmondson, 2008)
9. Clear supported policies with consequences for violence	empowerment to set limits & manage aggression	
10. Work culture free from judgement or blame	↓ fear of failure & being perceived incompetent	Psychological safety (Samra, J, Gilbert, M. Shain, M., & Bilsker, 2012)
11. Consistent & timely violence follow-up; communication of VP actions after violence	↓ cynicism & normalization of violence	

References for supporting formal theory

Bakker, A. B., & Demerouti, E. (2007). The Job Demands-Resources model: state of the art. *Journal of Managerial Psychology*, *22*(3), 309–328. https://doi.org/10.1108/02683940710733115.

Bandura, A. (1982). Self-Efficacy Mechanism in Human Agency. *American Psychologist*, *37*(2), 122–147.

Canadian Resource Centre for Victims of Crime. (2005). *The Impact of Victimization*. Retrieved from https://www.crcvc.ca/docs/victimization.pdf

Edmondson, A. (1999). Psychological Safety and Learning Behavior in Work Teams. *Source: Administrative Science Quarterly*, *44*(2), 350–383.

Edmondson, A. C. (2008). Managing the Risk of Learning: Psychological Safety in Work Teams. In *International Handbook of Organizational Teamwork and Cooperative Working* (pp. 255–275). https://doi.org/10.1002/9780470696712.ch13

Samra, J, Gilbert, M. Shain, M., & Bilsker, D. C. for A. R. in M. H. and A. (CARMHA). (2012). What is Psychological Health & Safety. *Guarding MInds @ Work*, pp. 1–2. Retrieved from http://www.guardingmindsatwork.ca/docs/What_is_Psychological_Health_and_Safety.pdf

Table S4. Included documents.

Author(s)	Year	Title	Publication
Adams, J., Knowles, A., Irons, G., Roddy, A., & Ashworth, J	2017	Assessing the effectiveness of clinical education to reduce the frequency and recurrence of workplace violence	Australian Journal of Advanced Nursing
Arnetz, J. E., & Arnetz, B. B.	2000	Implementation and evaluation of a practical intervention programme for dealing with violence towards health care workers	Journal of Advanced Nursing
Ashton, R. A., Morris, L., & Smith, I.	2018	A qualitative meta-synthesis of emergency department staff experiences of violence and aggression	International Emergency Nursing
Baig, L., Tanzil, S., Shaikh, S., Hashmi, I., Khan, M. A., & Polkowski, M.	2018	Effectiveness of training on de-escalation of violence and management of aggressive behavior faced by health care providers in a public sector hospital of Karachi	Pakistan Journal of Medical Sciences
Banerjee, A., Daly, T., Armstrong, H., Armstrong, P., Lafrance, S., & Szebehely, M.	2008	Out of control: Violence against personal support workers in long-term care	Report (CIHR Funded Project)
Banerjee, A., Daly, T., Armstrong, P., Szebehely, M., Armstrong, H., & Lafrance, S.	2012	Structural violence in long-term, residential care for older people: Comparing Canada and Scandinavia	Social Science & Medicine
Beech, B., & Leather, P.	2006	Workplace violence in the health care sector: A review of staff training and integration of training evaluation models	Aggression & Violent Behaviour
Björkdahl, A., Hansebo, G., & Palmstierna, T.	2013	The influence of staff training on the violence prevention and management climate in psychiatric inpatient units	Journal of Psychiatric & Mental Health Nursing
Blando, J. D., O'Hagan, E., Casteel, C., Nocera, M. A., & Peek-Asa, C.	2013	Impact of hospital security programmes and workplace aggression on nurse perceptions of safety	Journal of Nursing Management
Blando, J., Ridenour, M., Hartley, D., & Casteel, C.	2014	Barriers to effective implementation of programs for the prevention of workplace violence in hospitals	Online Journal Of Issues In Nursing
Blouin, S.	2017	Taking a stand against workplace violence	Health Care Executive
Bond, P., Paniagua, H., & Thompson, A.	2009	Zero tolerance of violent patients: Policy in action	Practice Nursing
Brophy, J. T., Keith, M. M., & Hurley, M.	2018	Assaulted and Unheard: Violence Against Healthcare Staff	New Solutions: A Journal of Environmental And Occupational Health

Cassidy, E. L., Rosen, C., Cook, J., Greenbaum, M. A., Chow, H., Solano, N., & Sheikh, J. I.	2005	Assessment to intervention: Utilizing a staff needs assessment to improve care for behaviorally challenging residents in long term care (Part I)	Clinical Gerontologist
Chapman, R., Ogle, K. R., Martin, C., Rahman, A., Mckenna, B., & Barnfield, J.	2016	Australian nurses' perceptions of the use of manual restraint in the Emergency Department: A qualitative perspective	Journal of Clinical Nursing
Chen, W. C., Huang, C. J., Chen, C. C., & Wang, J. D.	2011	The incidence and risk factors of workplace violence towards female nurses reported via internet in an acute psychiatric hospital	Archives of Environmental & Occupational Health
Child, Rebekah J. Howerton, and Elizabeth J. Sussman.	2017	Occupational Disappointment: Why Did I Even Become a Nurse?	Journal of Emergency Nursing
Darling, F. E., Allen, D. E., de Nesnera, A., & Cummings, K.	2011	Transforming the culture of caring: Getting hurt is not part of the job	Journal of Psychosocial Nursing
Dickens, G., Rogers, G., Rooney, C., McGuinness, A., & Doyle, D.	2009	An audit of the use of breakaway techniques in a large psychiatric hospital: A replication study	Journal of Psychiatric and Mental Health Nursing
Duffin, C.	2010	Researchers question the effectiveness of training staff in breakaway techniques	Mental Health Practice
Duncan, S.M., Hyndamn, K., Estabrooks, C.A., Hesketh, K., Humphrey, C.K., Wong, J.S., Acorn, S. and Giovannetti, P.	2016	Nurses' experience of violence in Alberta and British Columbia hospitals	Canadian Journal of Nursing Research
Edmondson, A. C.	2008	Managing the risk of learning: Psychological safety in work teams	International Handbook of Organizational Teamwork and Cooperative Working
Farrell, G. & Salmon, P.	2010	Challenging behaviour: An action plan for education and training	Contemporary Nurse
Farrell, G., & Cubit, K.	2005	Nurses under threat: a comparison of content of 28 aggression management programs	International Journal of Mental Health Nursing
Forster, J. A., Petty, M. T., Schleiger, C., & Walters, H. C.	2005	kNOw workplace violence: developing programs for managing the risk of aggression in the health care setting	The Medical Journal of Australia
Gerdtz, M. F., Daniel, C., Dearie, V., Prematunga, R., Bamert, M., & Duxbury, J.	2013	The outcome of a rapid training program on nurses' attitudes regarding the prevention of aggression in emergency departments: A multi-site evaluation	International Journal of Nursing Studies
Gillespie, G. L., Farra, S. L., & Gates, D. M.	2014	A workplace violence educational program: a repeated measures study	Nurse Education in Practice
Gillespie, G. L., Gates, D. M., Kowalenko, T., Bresler, S., & Succop, P.	2014	Implementation of a comprehensive intervention to reduce physical assaults and threats in the emergency department	Emergency Nursing: JEN
Gillespie, G. L., Gates, D. M., Mentzel, T., Al-Natour, A., & Kowalenko, T.	2013	Evaluation of a comprehensive ED violence prevention program	Journal of Emergency Nursing
Gillespie, G. L., Gates, D. M., Miller, M., & Howard, P. K	2010	Workplace Violence in Healthcare Settings: Risk Factors and Protective Strategies	Rehabilitation Nursing
Halm, M.	2017	Aggression management education for acute care nurses-What's the evidence?	American Journal of Critical Care
Heckemann, B., Breimaier, H. E., Halfens, R. J. G., Schols, J. M. G. A., & Hahn, S.	2016	The participant's perspective: learning from an aggression management training course for nurses. Insights from a qualitative interview study	Scandinavian Journal of Caring Sciences
Henderson, L., Kamp, B., Niedbalski, K., Abraham, S. P., & Gillum, D. R.	2018	Nurses' Perspectives on Patient and Visitor Violence: A Qualitative Study	International Journal of Studies in Nursing
Hills, D.	2008	Relationships between aggression management training, perceived self-efficacy and rural general hospital nurses' experiences of patient aggression	Contemporary Nurse.

Ilkiw-Lavalle, O., Grenyer, B. F., & Graham, L.	2012	Does prior training and staff occupation influence knowledge acquisition from an aggression management-training program?	International Journal of Mental Health Nursing
Jussab, F., & Murphy, H.	2015	"I just can't, I am frightened for my safety, I don't know how to work with her": Practitioners' experiences of client violence and recommendations for future practice	Professional Psychology: Research & Practice
Kansagra, S.M., Rao, S.R., Sullivan, A.F., Gordon, J.A., Magid, D.J., Kaushal, R., Camargo, Jr, C.A. and Blumenthal, D.	2008	A survey of workplace violence across 65 US emergency departments	Academic Emergency Medicine
Koller, L. H.	2016	It could never happen here: promoting violence prevention education for emergency department nurses	Journal of Continuing Education
Lanctôt, N., & Guay, S.	2014	The aftermath of workplace violence among healthcare workers: A systematic literature review of the consequences	Aggression and Violent Behavior
Lanza, M., Schmidt, S., & Mcmillan, F., Demaio, J., & Forester, L.	2011	Support Our Staff-A Unique Program to Help Deal With Patient Assault.	Perspectives in Psychiatric Care
Leadbetter, D.	2007	Challenging behaviour: Whose problem is it?	Nursing & Residential Care
Lee, B., Del Rosario, K., & Byron-Iyamah, C.	2017	Incorporating Relationship-Based Care Into a Nurse Education Program for Managing Disruptive Patient Behaviors	Clinical Nurse Specialist CMS
Luck, L., Jackson, D., & Usher, K.	2008	Innocent or culpable? Meanings that emergency department nurses ascribe to individual acts of violence	Journal of Clinical Nursing
MacGabhann, L.	2002	Prevention and management of violence: Implementing clinically effective practice	Mental Health Practice
Marshall, B., Craig, A., & Meyer, A.	2017	Registered nurses' attitudes towards, and experiences of, aggression and violence in the acute hospital setting	Kai Tiaki Nursing Research
Martinez, A. J. S	2016	Managing Workplace Violence with Evidence-Based Interventions: a Literature Review	Journal of Psychosocial Nursing & Mental Health Services
Moylan, L.	2015	A Conceptual Model for Nurses Decision-making with the Aggressive Psychiatric Patient.	Issues in Mental Health Nursing
Needham, I., Abderhalden, C., Halfens, R. J., Fischer, J. E., & Dassen, T.	2005	Non-somatic effects of patient aggression on nurses: a systematic review	Journal of Advanced Nursing
O'Grady, E., Dempsey, L., & Fabby, C.	2012	Anger: a common form of psychological distress among patients at the end of life	International Journal of Palliative Nursing
Parish, C.	2013	Change ward culture to cut violence and aggression	Mental Health Practice
Paterson, B., Leadbetter, D., & Bowie, V.	1999	Supporting nursing staff exposed to violence at work	International Journal of Nursing Studies
Pich, J., Hazelton, M., Sundin, D., & Kable, A.	2010	Patient-related violence against emergency department nurses	Nursing & Health Sciences
Price, O., Baker, J., Bee, P., & Lovell, K.	2015	Learning and performance outcomes of mental health staff training in de-escalation techniques for the management of violence and aggression	British Journal of Psychiatry
Renker, P., Scribner, S. A., & Huff, P.	2015	Staff perspectives of violence in the emergency department: Appeals for consequences, collaboration, and consistency	Work
Rosenau, B.	2017	Violence Prevention and Nurse Safety	Canadian Nurse
Sato, K., Wakabayashi, T., Kiyoshi-Teo, H., & Fukahori, H	2013	Factors associated with nurses' reporting of patients' aggressive behavior: A cross-sectional survey	Journal of Nursing Management
Stevenson, K. N., Jack, S. M., O'Mara, L., & LeGris, J.	2015	Registered nurses' experiences of patient violence on acute care psychiatric inpatient units: an interpretive descriptive study	BMC Nursing
Stubbs, B., & Dickens, G.	2008	Prevention and management of aggression in mental health: an interdisciplinary discussion	International Journal of Therapy & Rehabilitation

Sturrock, A.	2010	Restraint in inpatient areas: the experiences of service users	Mental Health Practice
Tölli, S., Partanen, P., Kontio, R., & Häggman-Laitila, A.	2017	A quantitative systematic review of the effects of training interventions on enhancing the competence of nursing staff in managing challenging patient behaviour	Journal of Advanced Nursing
Vandecasteele, T., Van Hecke, A., Duprez, V., Beeckman, D., Debyser, B., Grypdonck, M., & Verhaeghe, S.	2017	The influence of team members on nurses' perceptions of transgressive behaviour in care relationships: A qualitative study	Journal of advanced nursing
Wang, S., O'Brien-Pallas, L. L., & Hayes, L.	2008	A review and evaluation of workplace violence prevention programs in the health sector	Nursing Health Services Research Unit
Whitman, E.	2016	Frequent violence in the ED doesn't have to be part of the job	Modern Healthcare
Zarola, A., & Leather, P.	2006	Violence and aggression management training for trainers and managers A national evaluation of the training provision in healthcare settings	Report prepared for the Health and Safety Executive, Nottingham

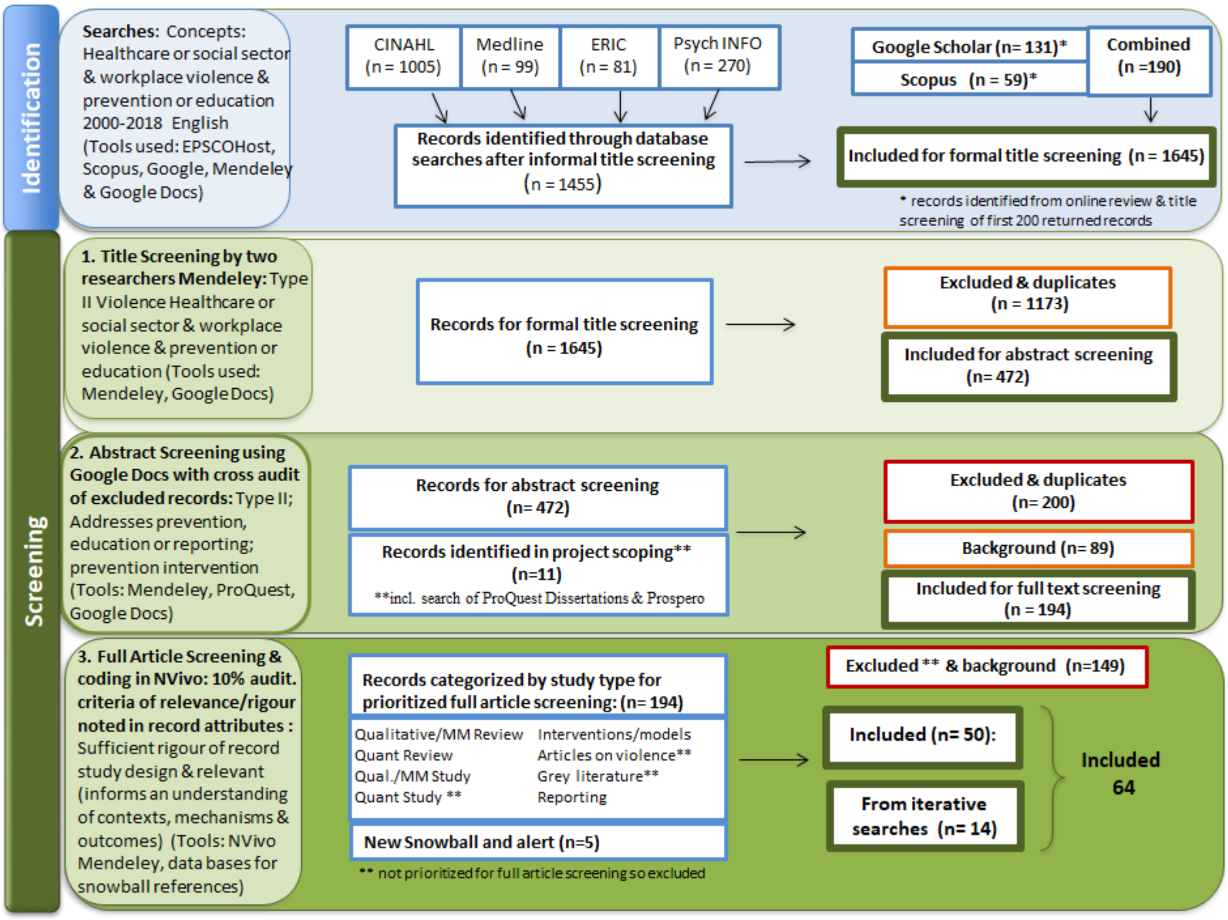

Figure S1: Search and screening strategy

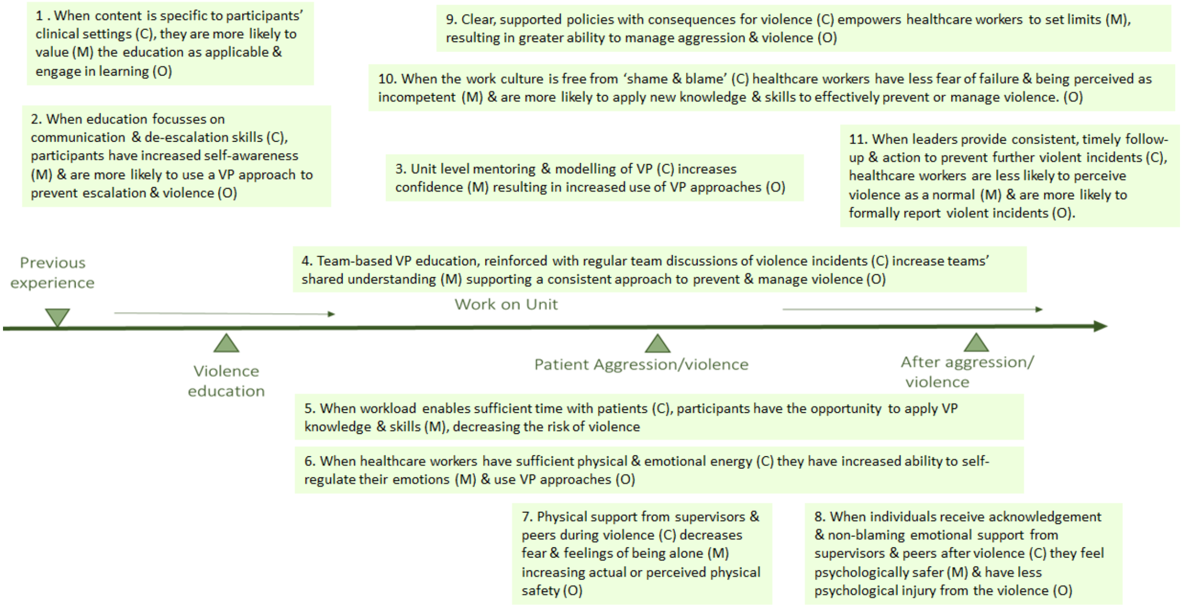

Figure S2 Review refined program theory
